# Cow’s Milk in Human Nutrition and the Emergence of Plant-Based Milk Alternatives

**DOI:** 10.3390/foods12010099

**Published:** 2022-12-25

**Authors:** I. C. Antunes, R. Bexiga, C. Pinto, L. C. Roseiro, M. A. G. Quaresma

**Affiliations:** 1 CIISA–Centre for Interdisciplinary Research in Animal Health, Faculty of Veterinary Medicine, University of Lisbon, 1300-477 Lisbon, Portugal; 2 Associate Laboratory for Animal and Veterinary Sciences (AL4AnimalS), 1300-477 Lisbon, Portugal; 3 Faculdade de Ciências Agrárias e do Ambiente da Universidade dos Açores, Rua Capitão João d’Ávila, 9700-042 Angra do Heroísmo, Portugal; 4 Food Technology and Safety Division, National Institute for Agricultural and Veterinary Research (INIAV, IP), Quinta do Marquês, 2780-157 Oeiras, Portugal

**Keywords:** cow’s milk, plant-based milk alternatives, nutritional composition, health issues

## Abstract

Cow’s milk is considered a complete food, providing high-quality protein and essential micronutrients, including vitamins and minerals. For medical reasons or as a lifestyle choice, consumers are replacing cow’s milk with plant-based milk alternatives (PBMA); some perceive them as healthier alternatives to cow’s milk due to their low saturated fatty acid content and no cholesterol content. However, the nutritional composition of PBMA is quite variable between different types and even within, which makes a comparison with cow’s milk a complex issue. Furthermore, the consumption of PBMA has been associated with the development of some diseases in infants and children. Meanwhile, the consumption of cow’s milk in human health is a controversial issue since it has been associated with a favorable effect in some diseases (such as obesity, type 2 diabetes, and Alzheimer’s) and a negative effect in others (such as prostate cancer risk and Parkinson’s disease); while in some diseases, there is no consensus in the cow’s milk consumption effect. The aim of this review is to make a nutritional comparison of cow’s milk with PBMA and to clarify the potential health issues related to their consumption.

## 1. Introduction

Cow’s milk is consumed daily by billions of people worldwide [1] and is recognized as a complete food. Cow’s milk provides both macro and micronutrients essential to the growth and development of the human body [2]. Its protein is considered to be of high biological value, presenting all essential amino acids and having high digestibility [3]. Despite its nutritional composition, the role of milk and dairy products in human nutrition has been increasingly questioned and studied among the scientific community [4], revealing that milk and dairy consumption may have a protective role against most prevalent chronic diseases, and very few adverse effects have been reported [5].

Due to health consequences (such as allergy to cow’s milk and lactose intolerance) or because of lifestyle choices (such as veganism and vegetarianism), consumer demand for cow’s milk alternatives has increased, leading to a rise in the market shares of plant-based milk alternatives (PBMA) and an increase in the varieties of PBMA [6,7]. Some consumers perceive PBMAs as healthy alternatives to cow’s milk [7]. The negative image of cow milk’s health is primarily associated with the lipid fraction composition, which has a high proportion of saturated fatty acids (SFA) and considerable amounts of cholesterol [6,8]. In contrast, all PBMA types are absent of cholesterol, and most have low SFA and high unsaturated fatty acid contents (with the exception of coconut PBMA) [5,9]. However, some authors have expressed concerns that the nutritional composition of PBMA is quite variable between different types, and even within a single type, when considering different brands. Such variability is dependent on raw material composition, processing methodology, and fortification processes [5,8].

The overall nutritional composition of PBMA is dependent on many variables, and there is also a concern that homemade PBMA (without nutritional fortification) may present a deficient composition. Moreover, PBMAs are also associated with some health concerns since soya PBMA may represent a source of isoflavones, which are known to display estrogen-like effects (as reviewed in Section 4.1), while rice PBMA may present a high inorganic arsenic content [5].

The aim of this review is to present an unbiased nutritional comparison of cow’s milk with different PBMA types, considering macro (carbohydrate, protein, and lipid fractions) and micronutrient (minerals and vitamins) compositions, and to clarify the potential health issues related to the consumption of these beverages. 

## 2. Nutritional Comparison of Cow’s Milk and PBMA

### 2.1. What Is A PBMA, and How Is It Made?

PBMAs are water extracts from cereals (e.g., oat or rice), pseudo-cereals (e.g., quinoa or amaranth), legumes (e.g., soya or peanut), nuts (e.g., almond, coconut or hazelnut), and seeds (e.g., sesame or flax) that are used as replacements for cow’s milk [10]. In the past, the term milk was used to designate these PBMAs and establish a sense of similarity between cow’s milk and PBMA among consumers [7]. The labeling of PBMA is a controversial issue since, in some countries, the use of the word “milk” is allowed, while in others, it is forbidden. In Europe, the Council Regulation (EC; No 1234/2007 of 22 October 2007) established that “the term ‘milk’ means exclusively the normal mammary secretion obtained from one or more milkings without either addition there to or extraction there from” [11]. However, this regulation includes two exceptions, which have no link to PBMA: 1) coconut milk and 2) almond milk [12]. In the United States of America, the Food and Drug Authority has defined milk as “the lacteal secretion, practically free from colostrum, obtained by the complete milking of one or more healthy cows” [13]. Furthermore, India’s law does not allow the use of the word “milk” in PBMA labeling [14], while Canadian law considers PBMA as a “beverage” and not “milk” [15]. On the other hand, in Australia, the label “soy milk” is allowed [16].

The technology of PBMA production is beyond the scope of this review; nevertheless, there are some important considerations to address. PBMA production makes use of simple methodologies commonly used in food production, including some industrial secrets. PBMAs are made from a wide variety of raw materials, which may require a unique processing protocol. Still, some major steps are common to different PBMA types and will be briefly discussed. Depending on the raw materials, some require pretreatment to improve rheologic and nutritional properties. The extraction is an important phase of PBMA production since it affects the final product’s characteristics. To obtain the water extracts, milling can be performed directly on dried materials (followed by extraction in water) or after soaking in water (wet milling). Depending on the PBMA, the extraction can be optimized by increasing pH or temperature or by enzyme addition. Thereafter, filtration, decantation, or centrifugation has to be performed, and several additives are required, including emulsifiers, stabilizers, flavors, sweeteners, and nutritional supplementation with vitamins and minerals (sufficiently stable and preferably bioavailable). Furthermore, homogenization will be required to improve the suspension stability, and finally, a thermal treatment (usually pasteurization or ultra-high pasteurization) is applied to assure microbiology stabilization [17,18]. Therefore, the nutritional composition of PBMA is not only dependent on the raw material, but also on other factors, such as the number of dilutions of the vegetal extract, the processing, supplementation, and product formulation, resulting in differences in nutritional composition between PBMA types and brands [8,19].

### 2.2. Carbohydrates

Carbohydrates are a group of substances that can be classified according to their degree of polymerization, including monosaccharides, when only a single monomeric unit is present—such as glucose, fructose, and galactose; disaccharides, when two monomeric units are linked—such as lactose, sucrose, maltose, polyols, and sugar alcohols (sorbitol, mannitol, and xylitol); oligosaccharides, when three to nine monomeric units are bound by glycolytic bonds—such as maltodextrins and raffinose; or polysaccharides when ten or more monomeric units are bound by glycosidic bonds (for example, starch) [20,21]. Dietary carbohydrates play an important role in human health and nutrition, being the prime source of energy for the human body. They also play other roles in the human body, such as in lipid metabolism and colonic function through fermentation [20,21].

Sugars can be naturally present in food or can be added. Food supplementation with carbohydrates is a common practice, used to improve palatability or food preservation or to add functional properties [22]. However, the information on food labels does not distinguish between added sugars or sugars naturally present, presenting only total sugars per serving [22,23]. Therefore, the term “total sugar content” stated in the text reflects the total sugars per serving, according to the regulation of the European Union [23].

#### 2.2.1. Total Energy Content

Rice PBMA (54.2 kcal) and almond PBMA (24.3 kcal) showed the highest and lowest total energy contents, respectively, while the remaining beverages had values in between (Table 1). Although coconut PBMA presented the same total energy content as cow’s milk (54.0 kcal), this beverage showed the highest variability between studies, ranging from 19.0 kcal to 178.0 kcal. This situation may rely on differences in the number of dilutions used during PBMA processing between brands [19].

#### 2.2.2. Total Carbohydrate Content

The total carbohydrate content of PBMA is quite variable, ranging from 1.95 to 10.82 g/100 mL (Table 1). Rice and oat PBMA present the highest total carbohydrate contents (10.8 and 8.99 g/100 mL, respectively), whereas soya, coconut, and almond PBMA present the lowest total carbohydrate contents (2.94, 2.28, and 1.95 g/100 mL, respectively). Furthermore, there seems to be a considerable variation in total carbohydrate content between beverages of the same type: in oat PBMA, which presents the highest variability, total carbohydrate content varies between 3.33 g/100 mL and 18.8 g/100 mL. The remaining PBMAs present a variability between 3.03 and 6.88 g/100 mL. All the differences mentioned above may be explained by differences in raw material, product formulation, fortifiers and additives, and the number of dilutions of the vegetable extract [6,19]. Cow’s milk presents a total carbohydrate content of 4.88 g/100 mL and the lowest variability between studies (0.45 g/100 mL).

#### 2.2.3. Fiber Content 

Regarding fiber, the highest content is shown by oat PBMA (1.27 g/100 mL) (Table 1). This PBMA also presents the greatest variability between studies, ranging from 0.40 to 4.70 g/100 mL. Almond PBMA presents the second highest variability (0.27–1.60 g/100 mL) and the same fiber content as soya PBMA (0.52 g/100 mL). Coconut PBMA presents a variability between studies (0.32 g/100 mL) close to soya PBMA (0.40 g/100 mL) and 0.34 g/100 mL of fiber content. Finally, rice PBMA presents the lowest fiber content (0.10 g/100 mL), whereas fiber is not present in cow’s milk.

#### 2.2.4. Total Sugar Content and Sugar Profile 

The total sugar content of PBMA is variable between beverages, for example, oat (5.61 g/100 mL) and rice (5.13 g/100 mL) PBMA present more than twice the sugar content of soya PBMA (2.23 g/100 mL), and more than three times the content of almond (1.82 g/100 mL) and coconut (1.62 g/100 mL) PBMA (Table 1). The high sugar content of oat and rice PBMA may be the result of starch hydrolysis during processing, which results in high amounts of maltose and/or glucose [6]. Such a suggestion is sustained by the high maltose contents observed in both oat and rice PBMA (3.05 g/100 mL and 3.37 g/100 mL, respectively) and the high glucose content detected in rice PBMA (2.43 g/100 mL). Conversely, almond and soya PBMA reveal higher contents of sucrose (1.80 and 1.13 g/100 mL, respectively) and fructose (0.34 and 0.78 g/100 mL, respectively), which can be the result of adding sugars to the formula, such as apple concentrate, sucrose, or maple syrup, which are high in fructose or sucrose [6].

Cow’s milk presents a sugar content (4.81 g/100 mL) lower than rice and oat PBMA. Lactose is the natural and exclusive sugar of cow’s milk. This disaccharide is composed of glucose and galactose linked by a β-1,4 glycosidic bond, which is hydrolyzed by the enzyme lactase (β-galactosidase) since mammals cannot absorb disaccharides [3]. Regular milk has mainly lactose in its sugar composition [6], whereas lactose-free milk includes hydrolyzed lactose, i.e., glucose (2.06 g/100 mL) and galactose (2.55 g/100 mL).

#### 2.2.5. Glycemic Index (GI) and Glycemic Load (GL) 

To quantify the effect of food consumption on blood glucose concentration, both GI and GL are used. Glycemic index represents the relative rise of glucose concentration in blood (compared with a reference carbohydrate) after ingestion of a single food containing 50 g of carbohydrates, while GL represents the GI and the quantity of ingested food [36]. Glycemic index can be divided into three categories: GI values below 40 are defined as low (using white bread as the reference); GI values between 40 and 70 are defined as medium; and GI values above 70 are defined as high [36]. Most foods with a high GI also have high GL (when consumed in standard serving size); however, some foods with a moderate GI can also generate a high GL when consumed in excess. Rice PBMA and coconut PBMA present the highest GI values (GI of 99 and 97, respectively), similar to the values present by maltose (GI of 105), glucose (GI of 99), Chocapic® (GI of 84), and cornflakes (GI of 81) (Table 2). While oat, almond, and soya PBMA present intermediate GI values (GI of 60, 57, and 53, respectively), similar to the GI values presented by Coca-Cola® (GI of 58), orange juice (GI of 50), muesli (GI of 66), Weetabix® (GI of 55), honey (GI of 55), and sucrose (GI of 68). Finally, cow’s milk presents the lowest GI value (GI of 47), which is similar to lactose’s GI value (GI of 46). Although oat PBMA has a high carbohydrate content, oats’ β-glucan content reduces the GI value [37] to a moderate level (GI of 60) [6]—an example of the glycemic response not only being influenced by the total amount of the carbohydrates per se [38]. Likewise, cow’s milk presents a high carbohydrate content compared with some PBMA but shows a moderate value of GI [6]. The low rise of blood glucose after cow’s milk ingestion can be a consequence of its high protein content [3,39] since the ingestion of milk’s proteins is known to reduce blood glucose response [40]. Östman et al. [41] demonstrate that insulin secretion can be stimulated by other food factors since the consumption of milk products generate greater insulinemia than the ingestion of equivalent amounts of lactose.

Regarding GL, rice PBMA has the highest value (GL of 18), which is very close to the value present by some breakfast cereals (GL of 17–21) and Coca-Cola® (GL of 15) (Table 2). The other PBMAs present lower GL values (GL of 2–8), as does cow’s milk (GL of 4). These GL values are similar to those in some sugars and sugar alcohols (GL of 2–11). 

### 2.3. Protein 

The twenty main or proteinogenic amino acids are grouped into two major groups—essential or indispensable if the body cannot synthesize them from precursors and they must be provided by the diet; and non-essential or dispensable, if the body synthesizes them in adequate amounts [43,44]. Nevertheless, in some conditions, either physiologic or pathologic, when the body cannot synthesize specific amino acids in adequate amounts, the amino acids can be classified as conditionally essential amino acids [44]. Dietary protein is a source of nitrogen for all nitrogenous compounds within the human body [43,45].

#### 2.3.1. Total Protein Content

The protein fraction of cow’s milk is, on average, 3.35 g/100 mL (Table 1), and it is mainly composed of two groups—caseins and whey proteins [8,46]. The protein fraction of PBMA is variable between types [8,25]. Soya PBMA presents the highest protein content among PBMA beverages (3.10 g/100 mL), while the other PBMA types present either a low content, as in oat PBMA (1.10 g/100 mL), or very low protein content, as in almond, coconut, and rice PBMA (ranging between 0.30 and 0.75 g/100 mL). Moreover, cow milk protein content has a low variation between studies (range from 3.15 g/100 mL to 3.70 g/100 mL) as opposed to the high variability observed in PBMA, which occurs between different types and within the same type [19].

#### 2.3.2. Protein Quality Evaluation

The evaluation of dietary protein quality focuses on the ability of a food to provide nitrogen and amino acids for growth and maintenance [43,45]. Such quality is dependent on several variables, such as digestibility, amino acid profile, and bioavailability [8]. Table 3 gathers the true ileal amino acid digestibility of different protein sources. Whey protein isolate has the maximum digestibility (100%), followed by whey protein concentrate (98%) and milk protein concentrate (94%). Pea protein concentrate (98%) and soya protein isolate (96%) are the only plant protein sources with a digestibility value close to milk protein digestibility. The remaining plant protein sources show a lower digestibility value (ranging between 77 and 93%). The differences in digestibility values may be explained by internal (e.g., amino acid profile, protein folding, and crosslinking) and external factors (e.g., food processing, presence of emulsifiers, and/or antinutritional factors) [47]. In fact, many foods present high levels of antinutritional factors such as tannins, phytates, trypsin inhibitors, or glucosinolates that occur naturally in the source [48]. Furthermore, according to their amino acid profile, dietary proteins are traditionally divided into complete and incomplete proteins. Complete proteins offer all the essential amino acids, while incomplete proteins fail to provide some essential amino acids such as methionine, cysteine (e.g., leguminous, or lysine (e.g., cereals) [8,9].

The protein digestibility-corrected amino acid score (PDCAAS) is the protein quality measure recommended to assess dietary protein quality [45]. However, the PDCAAS is associated with protein quality overestimation of low-quality protein sources (e.g., plant proteins) and protein quality underestimation of high-quality protein sources (e.g., dairy proteins) due to PDCAAS truncation at 100 [49]. These limitations have led some authors to recommend the use of the digestible indispensable amino acid score (DIAAS) instead of PDCAAS to measure protein quality [48,51]. According to FAO [48], foods presenting DIAAS values ≥ 100 can be classified as “excellent/high” protein sources, whereas foods presenting DIAAS values of 75–99 can be classified as “good” sources of protein. No nutritional claims should be made for protein sources presenting DIAAS values below 75. In their work, Mathai et al. [51] made a comparison of both protein quality assessment scores (DIAAS and PDCAAS) and found that dairy protein sources show PDCAAS and DIAAS values higher than plant protein sources (Table 4). Rutherfurd et al. [33] also demonstrate that dairy protein sources have higher DIAAS values than plant protein sources; however, these authors use a different amino acid requirement pattern and animal model, which may explain why some of the DIAAS values are lower than those shown by Mathai et al. [51]. 

### 2.4. Lipids

Fats and oils represent a large number of lipid compounds, including fatty acids, mono-, di-, and triacylglycerols, phospholipids, and sterols [52]. Fatty acids are used as building blocks of both triacylglycerols and phospholipids and are also used in the synthesis of signaling molecules (eicosanoids) [53,54]. 

#### 2.4.1. Total Cholesterol Content

Cholesterol is a structural component of cellular and subcellular membranes, helping to create a semi-permeable barrier between cellular compartments. It also participates in transmembrane signaling processes, regulates membrane fluidity, and is used as a substrate for the synthesis of other steroid molecules, such as vitamin D, bile acids, and steroid hormones [55,56]. Humans obtain cholesterol through liver biosynthesis or through the diet [56,57]. 

The total cholesterol content of cow’s milk varies based on total fat content; therefore, whole milk has a higher cholesterol content (10 mg/100 mL) than semi-skimmed milk (8 mg/100 mL) or skimmed milk (2 mg/100 mL) [27]. Because it is a sterol exclusive to animal cells [53], there is no cholesterol in PBMA.

#### 2.4.2. Total Lipid Content

The total lipid content of cow’s milk averages 2.81 g/100 mL (Table 1). However, this value can be much lower in semi-skimmed (1.98 g/100 mL) and skimmed milk (0.08 g/100 mL) [27]. Therefore, the variability observed in cow’s milk (0.08–4.17 g/100 mL) is the result of the skimming process. On the other hand, PBMAs are quite different in terms of total lipid contents, presenting a great variability between beverages (ranging from 0.96 to 4.55 g/100 mL) and within the same beverage type. For example, coconut PBMA presents the highest total lipid content (4.55 g/100 mL), a superiority of 61.9% relative to whole milk, and the greatest variation between studies (ranges from 0.84 g/100 mL to 18.5 g/100 mL). These results may be explained by the number of dilutions in water that coconut milk is submitted to when producing coconut PBMA [8]. Therefore, there are beverages on the market with higher or lower total lipid contents for both milk and PBMA. The total lipid content is of importance regarding its contribution to the caloric value, but its fatty acid profile is also of great importance due to biologic functions [53,58].

#### 2.4.3. Fatty Acid Profile

Milk lipids occur mainly as triacylglycerols (98%), which are composed of fatty acids with different carbon chain lengths and saturation levels [3]. Over 400 fatty acids occur in cow’s milk [59], but only 15 fatty acids present a proportion similar to or above 1%, whereas the remaining fatty acids are present in trace amounts [60]. The fatty acid composition of cow’s milk depends on (1) synthesis de novo in the mammary gland; (2) gastrointestinal absorption from digestion; and (3) mobilization from body fat reserves [59,61], and can be influenced by the animals’ feed, stage of lactation, breed, and season [59,60]. Saturated fatty acids (SFA) are the main group of fatty acids (ranging between 57.0 and 70.0% of total fatty acids) in cow’s milk fat, whereas monounsaturated fatty acids (MUFA; ranging between 24.8 and 29.0% of total fatty acids) and polyunsaturated fatty acids (PUFA; ranging between 3.7 and 6.0% of total fatty acids) are present in lower concentrations. 

The high SFA content of cow’s milk is a negative attribute. However, the hypercholesterolemic effect associated with SFA and the consequent rise in low-density lipoprotein (LDL) cholesterol is related to three fatty acids, namely C12:0, C14:0, and C16:0 (lauric, myristic, and palmitic acids, respectively) [52,59]. Together, these three fatty acids represent, on average, 63.5% of milk SFA [58]. Stearic acid (C18:0), also belonging to SFA, is considered neutral since it does not increase LDL cholesterol. On the other hand, oleic acid, the main fatty acid of the MUFA group, together with PUFA, lowers LDL cholesterol concentration [52,62]. In the PUFA group, linoleic acid (C18:2 n-6) and α-linolenic acid (C18:3 n-3) are considered essential fatty acids since they cannot be synthesized by the body [52]. α-linolenic acid is the precursor of eicosapentaenoic acid (EPA; C20:5n-3) and docosahexaenoic acid (DHA; C22:6n-3), which are the most important n-3 fatty acids in human nutrition. These fatty acids are essential for brain and retina development and function, particularly in premature infants [52]. Moreover, EPA and DHA are precursors of eicosanoids, such as prostaglandin (PG) E3, thromboxane (TX) A3, prostacyclin 3 (PGI), and leukotriene (LT) B5, which are known for their health benefits (anti-atherogenic, anti-thrombotic, and anti-inflammatory) in cardiovascular diseases and inflammatory diseases, such as rheumatoid arthritis [52,63,64]. 

*Trans* fatty acids (TFA), mainly composed of isomers of oleic acid, are known to increase total blood and LDL cholesterol [53], which implicate these fatty acids in the development of heart disease [59]. An FAO report states that there is convincing evidence that TFA increases coronary heart disease (CHD) risk factors and events. However, in the human diet, TFA intake comes mainly from partially hydrogenated vegetable oils and not from animal sources since, in most societies, the average daily consumption of ruminal-originated TFA is low [52]. In cow’s milk, TFA represents 3.2% of total fatty acids, where vaccenic acid (C18:1, *trans*-11) is the predominant isomer, representing 46.5% of total TFA [58]. In mammals, by the action of ∆9 desaturases, vaccenic acid can be partially converted into rumenic acid (C18:2,*cis*-9,*trans*-11), which is the most representative isomer of conjugated linoleic acid (CLA) and known for anti-carcinogenic, anti-atherogenic, anti-diabetic properties, and improvement of immune function [3,53].

Regarding PMBA, the coconut PBMA fatty acid profile is mainly composed of SFA (averaging 89.2% of total fatty acids). In this SFA fraction, the predominant fatty acids are C12:0, C14:0, and C16:0 since coconut PBMA is made from coconut milk dilution, which is mainly composed of these fatty acids [8,65]. As stated before, these fatty acids are a matter of concern since they are known to have negative implications on human health [52]. The fatty acid profile of almond, oat, and rice PBMA is predominated by MUFA (averaging 64.8% of total fatty acids, 61.6% of total fatty acids, and 56.3% of total fatty acids, respectively), while in soya PBMA, PUFAs are the predominant fatty acids (55.2% of total fatty acids).

### 2.5. Minerals

Minerals can occur in different chemical forms or as a part of other molecules (i.e., proteins, fats, carbohydrates, and nucleic acids). They play a range of roles in metabolic and physiological pathways in the human body, and the deficiency or excess of a single element can lead to several health issues in humans, being more severe as the amplitude of the deficiency/toxicity increases [66,67].

#### 2.5.1. Macromineral Contents

Macrominerals are minerals that present a concentration in the human body above 0.01% by weight [68]. Table 5 presents the macromineral composition of cow’s milk and PBMAs. The macrominerals include calcium, magnesium, sodium, potassium, and phosphorus. 

Potassium and calcium (144.2 and 121.2 mg/100 mL, respectively) are the macrominerals present in the highest concentrations in cow’s milk. Between PBMAs, soya PBMA (157.4 mg/100 mL) has the highest potassium content, while the remaining PBMAs show much lower potassium contents (62.4 g/100 mL for almond PBMA, 60.2 g/100 mL for coconut PBMA, and 37.1 g/100 mL for oat and rice PBMAs). On the other hand, calcium is the macromineral in the highest concentration in almond PBMA (156.4 g/100 mL) and oat PBMA (114.1 g/100 mL), with the remaining PBMAs containing lower concentrations (90.8 g/100 mL for rice PBMA, 74.7 g/100 mL for soya PBMA, and 65.5 g/100 mL for coconut PBMA). Regarding phosphorus, cow’s milk has the highest phosphorus content (84.4 mg/100 mL), whereas rice PBMA (39.46 mg/100 mL) and soya PBMA (38.1 mg/100 mL) have less than half of that content. The remaining PBMA beverages (coconut, oat, and almond) have much lower phosphorus contents (24.67, 24.6, and 14.95 mg/100 mL, respectively). 

The calcium:phosphorus (Ca:P) ratio is an important nutritional ratio since their metabolisms are closely linked to bone, gut, and kidney levels [70,71,72]. During infancy, special attention should be paid to this ratio as it is a period of rapid skeletal growth, being advised that infant formula should present a Ca:P ratio between 1.1 and 2.0 [73]. In adults, it is not possible to define a dietary value for this ratio; however, it is known that the Ca:P ratio in bone is between 1.6 and 1.8 [70]. When considering the average values of calcium and phosphorus contents of each beverage present in Table 5, it is possible to calculate the Ca:P ratio of all beverages (data not shown). Cow’s milk has the lowest Ca:P ratio value (1.4), being the only one in the range of Ca:P values that is advised for infant formula and proper bone development [70,73]. Regarding PBMA, there is considerable variability between beverages, with soya PBMA (2.0) presenting the lowest ratio value, followed by rice PBMA (2.3) and coconut PBMA (2.7). Oat PBMA (4.6) and almond PBMA (10.5) present a value much higher than the recommended value. These values deserve special attention since it is known that an unbalanced Ca:P ratio may lead to negative consequences on bone health [74].

Regarding magnesium, soya PBMA (16.8 mg/100 mL) and coconut PBMA (15.1 mg/100 mL) present the highest concentrations of this macromineral, whereas other beverages present a lower (11.1 mg/100 mL for cow’s milk) or a much lower concentration (8.34 mg/100 mL for rice PBMA and 7.78 mg/100 mL for almond PBMA). In the human body, magnesium plays important roles, including bone health and the maintenance of the electrical potential of nervous tissues, as well as the permeability and electrical potential of cell membranes [3,75,76]. Magnesium deficiency can lead to hypocalcemia and hypokalemia, resulting in consequences at the neurological and cardiac level (such as hypertension) since this macromineral is responsible for changes in calcium metabolism and changes in the sodium:potassium (Na:K) ratio at the cellular level—through the sodium-potassium pump activated by Mg-ATP and the calcium pump, which regulate the flux of important cations for blood pressure regulation [76,77,78].

Almond PBMA has the highest concentration of sodium (64.2 mg/100 mL), followed by oat PBMA (46.9 mg/100 mL) and soya PBMA (46.04 mg/100 mL). Cow’s milk has a sodium concentration (44.1 mg/100 mL) in between PBMA sodium concentrations (ranging from 23.9 mg/100 mL to 64.2 mg/100 mL). Considering the average values of sodium and potassium contents of each beverage in Table 5, it is possible to calculate the Na:K ratio of all beverages (data not shown). Cow’s milk and soya PBMA have the lowest Na:K ratio (0.3), followed by coconut PBMA (0.4). Almond and rice PBMAs have a Na:K ratio of 1, whereas oat PBMA has a value of 1.3. The beverages with higher Na:K ratio values have higher concentrations of sodium and lower concentrations of potassium in their composition, which may have negative implications on blood pressure regulation.

Considering cow milk’s macromineral composition and the level of intake that is assumed to ensure nutritional adequacy (AI), it is estimated that an adult man consuming 100 mL of cow’s milk receives 16.2% of calcium, 3.2% of magnesium, 2.9% of sodium, 4,1% of potassium, and 15.4% of phosphorus of the AI [70,74,76,79,80]. However, these AI values can be different since the mineral fraction of cow’s milk is dependent on certain animal characteristics (breed, age, lactation phase, health status) and environments (season, feeding, and water composition) [68,81]. The form in which minerals are found in milk is also important since it may influence their absorption and bioavailability [82]. Furthermore, other milk components, such as lactose, lipids, and proteins, are known to improve mineral absorption (e.g., calcium) [83]. Regarding PBMA, almond PBMA makes a greater contribution to AI for calcium (AI = 20.9%) and sodium (AI = 4.28%), and soya PBMA provides a higher contribution to AI for magnesium (AI = 4.80%) and potassium (AI = 4.50%). During PBMA production, the addition of water by grinding or decantation may lead to some mineral loss [9]. Therefore, the higher values presented by some PBMAs may be the result of PBMA fortification with some micronutrients for the purpose of resembling cow’s milk micronutrient composition. However, the bioavailability of these added micronutrients is not known, which is a concern—particularly in children that consume these beverages [26]. Furthermore, since PBMAs are plant-based water extracts, they can comprise antinutritional factors in their composition, such as oxalates and phytates that interfere negatively with mineral absorption through the formation of insoluble complexes [3].

#### 2.5.2. Trace Element Contents

Trace elements are those minerals that present a concentration below 0.01% by weight in the human body [68]. The trace element composition of cow’s milk and PBMAs, present in Table 5, includes iron, copper, zinc, selenium, and manganese.

Coconut PBMA is the richest beverage in iron (0.62 mg/100 mL), while cow’s milk is the poorest beverage in this trace element (0.03 mg/100 mL). The remaining PBMAs have an iron content below 0.5 mg/100 mL. Coconut PBMA has the highest contents of copper (0.13 mg/100 mL) and manganese (0.25 mg/100 mL), followed by soya PBMA (0.11 and 0.14 mg/100 mL, respectively). The remaining beverages have much lower contents of copper (ranging from 0.01 to 0.03 mg/100 mL) and manganese (ranging from 0.002 to 0.07 mg/100 mL). Cow’s milk presents the highest content of zinc, whereas all PBMAs have lower contents, with coconut PBMA being the richest PBMA in zinc (0.30 mg/100 mL). Finally, it is only possible to collect information on cow’s milk and coconut PBMA selenium contents, being cow’s milk the richest beverage in this trace element (2.33 mg/100 mL).

When considering cow’s milk and the PBMA trace element composition and AI, the consumption of 100 mL of cow’s milk by an adult man has 0.5% of iron, 0.63% of copper, 5.6% of zinc, and 0.07% of manganese of the AI [84,85,86,87]. Coconut PBMA was the beverage with the greatest contribution to AI for iron, copper, zinc, and manganese (10.3%, 8.1%, 4.0%, and 8.3%, respectively).

### 2.6. Vitamins

Vitamins are essential compounds present in food in small amounts. The human body has limited synthesis capacity; therefore, they need to be provided by the diet [88]. They play a series of functions in the human body, and their low or high intake can lead to health issues, being more severe as the magnitude of deficiency/toxicity increases. Depending on their solubility, they can be classified as fat-soluble vitamins (A, D, E, and K) or water-soluble vitamins (C and complex B) [88,89]. The information regarding the fat-soluble and water-soluble vitamin contents of PBMA is scarce, which makes it difficult to perform a comparison to cow’s milk (Table 6). 

#### 2.6.1. Fat-Soluble Vitamin Contents

Fat-soluble vitamins play a diversity of important functions in the human body that are essential for several functions, from vision (Vitamin A) [90], to bone health (Vitamin D) [91], antioxidant activity (Vitamin E) [92], or blood clotting (Vitamin K) [93]. Because of their important roles in human physiology, the deficient ingestion of these vitamins may impair several functions. For example, a fat-free diet can have a negative effect on human health, leading to malabsorption of vitamins, which can culminate in vitamin deficiency since fat-soluble vitamins are absorbed and transported with fat and stored in the liver and adipose tissue [94].

The vitamin A content is very similar between the observed milk beverages, with cow’s milk presenting with a content of 155.3 IU/100 mL, whereas the PBMA presents with a content that ranges from 141.6 to 195.4 IU/100 mL. Regarding vitamin D and vitamin E contents, PBMA has higher values (ranging between 38.49 and 42.95 IU/100 mL and 1.25 and 1.60 mg/100 mL, respectively) than cow’s milk (22.8 IU/100 mL and 0.04 mg/100 mL, respectively). There is no information regarding the vitamin K content of PBMA, while cow’s milk has a content of 0.26 µg/100 mL. 

Considering these fat-soluble vitamin contents and AI, the daily consumption of 100 mL of cow’s milk by an adult man ensures 15.50% of vitamin A, 3.80% of vitamin D, 0.31% of vitamin E, and 0.37% of vitamin K for AI [90,92,93,95]. However, due to their higher contents of vitamin A, vitamin E, and vitamin D, some PBMAs make a greater contribution to AI than cow’s milk. For example, for vitamin A, almond PBMA, rice PBMA, and coconut PBMA contribute 58.62%, 18.53%, and 19.02% to AI, respectively. Regarding vitamin D, PBMA contributes to AI 6.42% to 7.16%, whereas for vitamin E all PBMAs (with the exception of coconut PBMA since there is no information available) contribute 9.62% to 12.85% to AI. The higher contents of vitamin A and vitamin D in PBMA may be a consequence of the fortification process during PBMA manufacturing or the PBMA production itself since this procedure includes different raw materials and formulation processes, which can explain the great variability between different PBMAs and within the same type of PBMA [5,8].

**Table 6:** Vitaminic composition (fat-soluble and water-soluble vitamins) of cow’s milk and some (oat, almond, soya, rice, and coconut) plant-based milk alternatives (PBMA). The data are presented by the range and the average in parenthesis.

		Cow’s Milk	Oat PBMA	Almond PBMA	Soya PBMA	Rice PBMA	Coconut PBMA
Fat−soluble vitamins	A ^1^	34.17–203.3 (155.3)	62.50–208.0 (141.6)	107.1–208.3 (195.4)	45.23–208.8 (154.2)	93.77–208.8 (185.3)	83.33–208.3 (190.2)
	D ^1^	4.00–51.67 (22.8)	42.00 (−)	3.23–62.50 (38.49)	2.60–75.0 (42.4)	2.90–75.0 (40.79)	4.07–62.50 (42.95)
	E ^2^	0.01–0.07 (0.04)	−	1.60 (−)	1.67 (−)	1.25 (−)	−
	K ^3^	0.20–0.30 (0.26)	−	−	−	−	−
Water−soluble vitamins	C ^2^	0.20–1.54 (0.87)	0.70 (−)	−	−	−	−
	B1 ^2^	0.04–0.05 (0.04)	−	−	0.03 (−)	−	−
	B2 ^2^	0.17–0.19 (0.18)	0.13–0.21 (0.18)	0.01–0.18 (0.08)	0.10–0.21 (0.17)	0.13–0.21 (0.17)	0.17 (−)
	B3 ^2^	0.08–0.09 (0.09)	−	−	0.12 (−)	−	−
	B6 ^2^	0.04 (–)	−	−	0.04 (−)	−	−
	B9 ^3^	5.00–5.15 (5.03)	−	8.00 (−)	10.13–14.00 (12.1)	10.13 (−)	8.00–10.0 (9.5)
	B12 ^3^	0.37–0.54 (0.47)	0.25 (−)	0.42–1.25 (0.97)	0.28–1.25 (0.76)	0.42–0.62 (0.55)	0.31–1.25 (1.03)
References		[8,24,26,27]	[8,26,31]	[8,24,26,32]	[8,24,26]	[8,26,28,96]	[8,24,26,35]

^1^ Expressed as IU/100 mL; ^2^ expressed as mg/100 mL; ^3^ expressed as µg/100 mL.

#### 2.6.2. Water-Soluble Vitamin Contents

Water-soluble vitamins also play important roles in the human body, participating in a wide range of functions such as collagen synthesis (vitamin C, ascorbic acid) [97], energy-yielding reactions (vitamin B1, thiamine) [98], redox metabolism (vitamin B2, riboflavin) [99], formation of NAD and NADP (vitamin B3, niacin) [100], amino acid and lipid metabolism (vitamin B5, pantothenic acid) [101], co-factor of enzymes (vitamin B6, pyridoxine; vitamin B7, biotin) [102,103], RNA and DNA synthesis (vitamin B9, folate) [104], and co-enzyme (vitamin B12, cobalamin) [105].

The vitamin C content of oat PBMA (0.70 mg/100 mL) is similar to the value present in cow’s milk (0.87 mg/100 mL), while there is no information regarding the vitamin C content of other PBMAs. When focusing on B complex vitamins, no information is found on the contents of pantothenic acid and biotin for PBMA. PBMAs present higher contents of folate (ranging from 8.0 µg/100 mL to 12.06 µg/100 mL) than cow’s milk (5.03 µg/100 mL). Similarly, for cobalamin, cow’s milk presents a lower content (0.47 µg/100 mL) than the other PBMAs (0.55–1.03 µg/100 mL) with the exception of oat PBMA (0.25 µg/100 mL). Soya PBMA has the same pyridoxine content as cow’s milk (0.04 µg/100 mL). In cow’s milk and soya PBMA, the contents of thiamine (0.04 µg/100 mL and 0.03 µg/100 mL, respectively) and niacin (0.09 µg/100 mL and 0.12 µg/100 mL, respectively) are very similar. Finally, the riboflavin content of cow’s milk is the same as oat PBMA (0.18 µg/100 mL), while soya, rice, and coconut PBMAs have the same content (0.17 µg/100 mL). Almond has the lowest content of riboflavin (0.08 µg/100 mL). 

The consumption of 100 mL of cow’s milk by an adult man, considering the water-soluble vitamin contents present above and AI, provides 0.97% of vitamin C, 3.67% of thiamine, 13.85% of riboflavin, 0.56% of niacin, 2.67% of pyridoxine, 2.01% of folate, and 11.75% of cobalamin [93,98,99,100,102,104,105]. Oat PBMA has the same contribution to AI (13.85%) as cow’s milk for riboflavin, and all PBMAs (with the exception of oat PBMA) make a greater contribution to AI for folate (ranging from 3.20% to 4.82%) and cobalamin (ranging from 13.75% to 25.75%). Likewise, in the water-soluble vitamins, as explained for the fat-soluble vitamins, certain levels of vitamins are the result of the fortification process that takes place during the PBMA manufacturing process, being also responsible for the existing variability between different PBMAs and within the same type of PBMA [5,8]. For example, cobalamin is a vitamin exclusive of foods from animal sources [106]; therefore, the presence of this vitamin in PBMA is clear evidence of adding vitamins during PBMA manufacture [8].

## 3. Health Issues Related to Cow’s Milk Consumption

### 3.1. Adverse Reaction to Cow’s Milk Consumption

#### 3.1.1. Cow’s Milk Protein Allergy

Cow’s milk protein allergy is the result of an immunological mechanism against cow’s milk proteins, which can be mediated by immunoglobulin E (IgE), not mediated by IgE, or a mix of both. This type of allergy is generally found in infants and children, being uncommon in adults. Skin, respiratory, and gastrointestinal reactions are common symptoms in the manifestation of cow’s milk protein allergy, and in the neonatal period, inadequate growth is a direct consequence. Due to gastrointestinal symptoms being similar to those shown by lactose intolerant people, confusion between cow’s milk allergy and lactose intolerance is very common [107].

#### 3.1.2. Lactose Intolerance

Lactose intolerance is one of the reasons why consumers are replacing cow’s milk with PBMA. Lactose intolerance is the result of lactase gene expression reduction. Such a condition reduces the amount of lactase present in the small intestine and, consequently, the activity of lactase [108]. Therefore, humans with low or absent levels of lactase activity cannot hydrolyze lactose in the small intestine. This condition is called primary lactase deficiency (PLD), and it is estimated to affect approximately 70% of the world’s population, with great variability between ethnic groups [108,109]. The undigested lactose reaches the large intestine, where it is metabolized by bacteria [108], producing a considerable amount of gas (e.g., hydrogen, carbon dioxide, and methane), and a large amount of water is drawn in due to lactose’s osmotic effect, resulting in symptoms such as abdominal pain, flatulence, and diarrhea [3]. The type and severity of these symptoms varies between subjects since lactose tolerance depends on the amount of lactose ingested, the quantity of lactase present in the small intestine, and the type of carrying food [108,109]. On the other hand, lactase deficiency can also be the consequence of small intestine diseases (such as acute gastroenteritis or chronic intestinal inflammation) that provoke intestinal epithelium injuries leading to different degrees of lactose maldigestion. This condition is known as secondary lactase deficiency [108,109].

#### 3.1.3. A1 β-Casein and A2 β-Casein

Caseins (CNs) are almost 80% of total milk proteins and can be divided into four different types: αs1-CN, αs2-CN, β-CN, and K-CN [8,110]. The β-CN type presents twelve different variants, with A1 and A2 being the most common [111]. These two genetic variants are distinguished only by a difference in one amino acid at position 67—where the A1 variant has histidine, the A2 variant has proline [111,112]. This difference has consequences in milk technological characteristics and human nutrition, with the A2 variant being more desirable than the A1 variant. The latter is due to technological benefits and improvements in milk digestibility [113]. Furthermore, the intestinal problems often associated with lactose intolerance may result from the consumption of milk with the A1 variant instead of the body’s inability to break down lactose [111].

### 3.2. Chronic Diseases

In recent years, the role of milk and dairy products in human nutrition has been increasingly questioned and studied among the scientific community [4], revealing that milk and dairy consumption may have a protective role against most prevalent chronic diseases and very few adverse effects have been reported [5].

#### 3.2.1. Obesity

In 2016, it was estimated that 13% of the world’s adult population was obese, while in 2019, it was estimated that 38.2 million children under the age of 5 years were affected by this condition [114]. Consumption of dairy products may be an important factor in controlling this condition due to their nutritional richness [115]. There are epidemiologic data supporting the beneficial effect of dairy product consumption on weight maintenance [116], with the consumption of whole milk being associated with lower odds of being overweight or obese [117]. Furthermore, in the context of energy-restricted diets, the high consumption of dairy products results in both body weight and fat mass loss [115,118]; however, no beneficial effect is found when no energy-restricted diets are applied [118,119].

#### 3.2.2. Cardiovascular Diseases

Cardiovascular diseases (CVD) are a major public health concern [120] as one of the main causes of death worldwide [121]. The intake of dairy products has been associated with an increased risk of CVD [5] due to their fat content and composition, dominated by the SFA fraction [122]. However, there is no consensus on the role of diets rich in SFA and CVD risk [123]. The daily consumption of 200 mL of milk is not associated with coronary heart disease (CHD) [124], but the same daily consumption of milk is responsible for a significant 7% lower risk of stroke [125]. Additionally, the consumption of milk and yogurt is associated with a reduction of 8% in the risk of elevated blood pressure.

#### 3.2.3. Diabetes

Diabetes is a chronic disease that affects around 422 million people worldwide and was responsible for 1.5 million deaths in 2019 [126]. This disease can be classified as Type 1 Diabetes (T1D), when the body produces an inadequate amount of insulin, and Type 2 Diabetes (T2D), when the body cannot use insulin efficiently, being the predominant diabetes type [127]. T2D can be prevented or delayed by a healthy diet, regular physical activity, and avoiding smoking [127]. Regarding diet, the consumption of dairy products has been shown to decrease the risk of T2D [128], which clearly indicates a beneficial effect of milk consumption on T2D [4].

#### 3.2.4. Bone Health

Osteoporosis is characterized by bone density reduction and, consequently, an increase in bone fragility [129]. The available evidence regarding the role of dairy products on bone health is contradictory. Some reports show no significant association between milk intake and the risk of hip fracture (at any age and in either gender) [130] or the reduced risk of osteoporosis [131]. Other reports show an inverse association between milk and dairy product consumption and osteoporosis, with an observed reduction in the osteoporosis risk of 22–37% by increasing the intake of dairy products and milk (every additional 200 g per day) [131].

#### 3.2.5. Cancer

Cancer is responsible for millions of deaths worldwide [132]. The association between dairy consumption and cancer has been extensively studied, however, the results are controversial. Dairy consumption has been found to decrease the risk of colorectal cancer [4,133,134] in both men and women [133]. A potential beneficial effect of milk consumption on bladder and gastric cancer risk has also been found [135,136], but this effect is variable between different geographical regions and even between different dairy products [135]. The same is not true for prostate cancer, where it is observed that a high intake of milk is related to an elevated risk of prostate cancer, the recurrence and progression of prostate cancer, as well as prostate cancer mortality [4]. An increase in the intake of milk by 100–200 g/day has been associated with a rapid increase in the risk of prostate cancer [137] and high consumption of whole milk can increase by 50% the mortality risk of prostate cancer [138]. On the other hand, there is no association or conclusive results about the role of dairy product consumption on lung cancer risk [139,140] or liver cancer risk [141]. Regarding breast and ovary cancer, there is no consensus about the role of dairy consumption on these cancer risks. For example, some studies indicate that there is no association between milk consumption (< 450 g/day) and breast cancer risk [142] and between milk/dairy product consumption and the risk of ovarian cancer [143,144]. However, when the intake of milk is higher, there is an increased risk of developing these types of cancer [142,145].

### 3.3. Neurological Diseases

Dementia is a syndrome that results in cognitive (thinking or behavior) function deterioration, and it is estimated to affect 50 million people worldwide, with 10 million new cases every year [146]. The most common form of the disease is Alzheimer’s disease (AD), which contributes to 60–70% of the cases [146]. Milk consumption has been inversely associated with the risk of cognitive disorders [147], such as AD [4]. However, the same is not valid for Parkinson’s disease (PD), with the risk of this disease being significantly increased by dairy product intake [4]. It is estimated that an increase in the intake of milk of 200 g/day results in a 17% increased risk of PD [148], although the mechanism underlying this association is not well understood [149].

## 4. Health Issues Related to PBMA Consumption

Consumers should be aware of the nutritional composition of PBMAs, namely their protein content and the bioavailability of added micronutrients during PBMA manufacture when they perform a complete replacement of cow’s milk by PBMA. This modification deserves special attention in infant and children’s diets since nutritional deficiencies may occur, namely in terms of vitamins, minerals, and proteins [8,26], resulting in risks to their health. In fact, there are several cases of disease manifestations in children resulting from PBMA ingestion. This happens due to parents diagnosing cow’s milk intolerance/allergy, parents’ beliefs that PBMAs are healthier alternatives than infant milk formula/regular milk for their infant and children, or because of a lifestyle choice [150,151,152]. Therefore, diseases such as Kwashiorkor and rickets have been associated with the consumption of rice PBMA [151,153,154], whereas the consumption of almond PBMA has been associated with metabolic alkalosis [155], rickets [150], scurvy [156], goiter [157], and development delay [156]. Lastly, the consumption of soya PBMA has been associated with rickets and development delay [151,158]. Therefore, the consumption of these beverages by infants and children should be contraindicated [151,152,159,160].

### 4.1. Isoflavones

Isoflavones found in some foods (including soya, red clover, and kudzu root) have a chemical structure similar to 17β-estradiol, being able to bind to estrogen receptors. The main source of isoflavones in the human diet is soya, which mostly contains genistein, daidzein, and glycitein isoflavones and their respective glycosides [161,162]. Isoflavones exhibit a complex interaction with the endocrine system, presenting both estrogen-like and anti-estrogenic effects, depending on which estrogen receptor (α or β) they bind to [163,164]. Due to this double effect on the endocrine system, some concerns regarding isoflavone’s estrogenic effect have arisen [163]. According to EFSA, in postmenopausal women, there is no increased risk of adverse effects on the mammary gland, thyroid function, or uterus with the consumption of food isoflavone supplements [161].

On the other hand, it has been claimed that isoflavones have an important role in human health, especially in bone mineral density (postmenopausal women), maintenance of normal blood LDL-cholesterol level, protection against oxidative damage (DNA, proteins, and lipids), reduction of vasomotor symptoms associated with menopause, and contribution to normal hair growth. However, due to insufficient evidence, it is not possible to establish a relationship between soya isoflavone consumption and these health associations [165,166].

## 5. Conclusions

Cow’s milk has been a part of the human diet for a long time; however, consumers are replacing cow’s milk with PBMAs, considering them good and viable alternatives to cow’s milk. As shown in this review article, PBMA does not resemble cow milk’s nutritional composition. In fact, the nutritional composition of PBMA is quite variable between types and even between brands of the same beverage. Most of these beverages (except for soya PBMA) present a low protein content. They also present high sugar and low fat contents, with PUFA and MUFA being the predominant groups of fatty acids in PBMA (except for coconut PBMA), while cow milk’s fatty acid profile is dominated by SFA. Regarding minerals and vitamins, some PBMAs present equal or even higher contents of these micronutrients than cow’s milk, which may be a consequence of the fortification process during manufacture. The effect of cow milk or dairy product consumption on certain aspects of human health is still under discussion, whereas other aspects show consensus. Due to their nutritional composition, dairy products present a favorable effect on obesity control, type 2 diabetes, and Alzheimer’s disease and decrease the risk of stroke and elevated blood pressure. Regarding bone health, breast cancer, and ovarian cancer, there is no consensus between studies about the role of milk and dairy product intake, while in colorectal, bladder, and gastric cancers, a positive effect of dairy product intake is found. On the other hand, no association is observed between dairy product consumption in lung and liver cancer, whereas an increased risk of prostate cancer and Parkinson’s disease is associated with dairy product consumption. Lastly, the total replacement of cow’s milk with PBMAs by infants and children should be avoided due to the lack of some nutrients, such as minerals and vitamins, resulting in a range of developmental diseases that may pose a threat to infant and children’s health.

## Figures and Tables

**Table 1:** Nutritional composition (energy expressed as kcal/100 mL and macronutrients expressed as g/100 mL) of cow’s milk and some (oat, almond, soya, rice, and coconut) plant-based milk alternatives (PBMA). The data is presented by the range and the average in parenthesis.

		Cow’s Milk	Oat PBMA	Almond PBMA	Soya PBMA	Rice PBMA	Coconut PBMA
Total energy content		34.0–65.8 (54.0)	29.0–103.0 (52.7)	15.2–40.2 (24.3)	32.0–58.0 (41.2)	47.0–60.0 (54.2)	19.0–178.0 (54.0)
Carbohydrates	Total content	4.58–5.33 (4.88)	3.33–18.8 (8.99)	0.20–4.40 (1.95)	0.20–7.08 (2.94)	9.17–12.2 (10.82)	0.42–5.00 (2.28)
	Fiber	–	0.40–4.70 (1.27)	0.27–1.60 (0.52)	0.40–0.80 (0.52)	0.10 (−)	0.10–0.42 (0.34)
	Sugar	3.38–5.15 (4.81)	0.42–10.9 (5.61)	0.05–4.58 (1.82)	0.10–6.25 (2.23)	2.50–7.02 (5.13)	0.00–3.75 (1.62)
Sugar profile	Maltose	−	2.75–3.34 (3.05)	−	0.66 (−)	2.41–4.88 (3.37)	1.05 (−)
	Sucrose	−	−	0.16–3.42 (1.80)	0.35–2.88 (1.13)	−	−
	Glucose	2.06 (−)	0.01 (−)	0.06–0.87 (0.38)	0.01–0.52 (0.33)	0.11–4.12 (2.43)	0.81 (−)
	Fructose	−	−	0.06–0.61 (0.34)	0.06–1.27 (0.78)	0.07–0.10 (0.09)	−
	Galactose	2.55 (−)	−	−	−	−	−
Protein		3.15–3.70 (3.35)	0.4–3.24 (1.10)	0.41–2.40 (0.75)	2.08–3.70 (3.10)	0.07–0.42 (0.30)	0.08–1.60 (0.64)
Lipids	Total content	0.08–4.17 (2.81)	0.28–2.65 (1.21)	1.04–10.4 (2.42)	1.25–2.11 (1.76)	0.83–1.20 (0.96)	0.84–18.5 (4.55)
	SFA ^1^	0.06–2.35 (1.45)	0.00–1.47 (0.35)	0.10–0.20 (0.15)	0.17–0.42 (0.27)	0.07–0.21 (0.16)	1.67–12.6 (3.15)
	MUFA ^2^	0.02–1.09 (0.66)	0.83 (−)	0.70–0.83 (0.79)	0.35 (−)	0.48–0.62 (0.57)	−
	PUFA ^3^	0.00–0.20 (0.12)	0.21–0.42 (0.32)	0.21–0.28 (0.23)	1.00 (−)	0.21–0.35 (0.26)	−
References		[6,8,10,24,25,26,27]	[6,8,10,17,25,26,28,29,30,31]	[6,8,10,17,24,26,28,29,30,32]	[6,8,10,17,24,25,26,28,29,30,33]	[6,8,10,17,24,25,26,28,30,34]	[6,8,10,24,26,28,29,35]

^1^ SFA= sum of saturated fatty acids; ^2^ MUFA= sum of monosaturated fatty acids; ^3^ PUFA= sum of polyunsaturated fatty acids.

**Table 2:** Glycemic index (GI) and glycemic load (GL) values of different types of food.

Food Group	Food Description	GI	GL
Plant-based milk alternatives ^1^	Almond	57	3
	Coconut	97	5
	Oat	60	8
	Rice	99	18
	Soya	53	2
Cow’s milk ^1^	Fresh milk, pasteurized, and homogenized	47	4
Other beverages ^2^	Coca-Cola	58	15
	Apple juice unsweetened	40	12
	Orange juice	50	13
Breakfast cereals ^2^	Chocapic (Nestlé, France)	84	21
	Cornflakes	81	21
	Muesli, NS (Canada)	66	17
	Alpen Muesli (Wheetabix, France)	55	10
Sugar and sugar alcohols ^2^	Fructose	19	2
	Glucose	99	10
	Honey	55	10
	Lactose	46	5
	Maltose	105	11
	Sucrose	68	7

^1^ Data adapted from [6]; ^2^ data adapted from [42];

**Table 3:** True ileal amino acid digestibility (%) of different protein sources.

Protein Source	True Ileal Amino Acid Digestibility (%)
Whey protein isolate ^1^	100
Whey protein concentrate ^1^	98
Milk protein concentrate ^1^	94
Pea protein concentrate ^1^	98
Soya protein isolate ^1^	96
Whole wheat ^2^	93
Oats ^2^	89
Buckwheat ^2^	88
Brown rice ^2^	85
Polished rice ^2^	77

^1^ Data adapted from [49]; ^2^ data adapted from [50].

**Table 4:** Protein digestibility-corrected amino acid score (PDCAAS) and digestible indispensable amino acid score (DIAAS) values from dairy and plant proteins. The first-limiting amino acid is presented in parenthesis.

	Protein Quality Assessment Scores	DIAAS	PDCAAS (Untruncated)
Protein Source	Whey isolate protein	125 (His)	122 (His)
	Whey protein concentrate	133 (His)	134 (His)
	Milk protein concentrate	141 (SAA)	142 (SAA)
	Skimmed milk powder	123 (SAA)	132 (SAA)
	Pea protein concentrate	73 (SAA)	84 (SAA)
	Soya protein isolate	98 (SAA)	102 (SAA)
	Soya flour	105 (SAA)	109 (SAA)
	Wheat	54 (Lys)	51 (Lys)

Data adapted from [51]. Note: SAA, sulfur amino acids (methionine + cysteine).

**Table 5:** Mineral composition (macrominerals and trace elements expressed as mg/100 mL) of cow’s milk and some (oat, almond, soya, rice, and coconut) plant−based milk alternatives (PBMA). The data are presented as the range and the average in parentheses.

		Cow’s Milk	Oat PBMA	Almond PBMA	Soya PBMA	Rice PBMA	Coconut PBMA
Macrominerals	Calcium	113–134 (121.2)	17.4–146 (114.1)	20.20–188.0 (156.4)	8.00–187.5 (74.65)	3.90–146.0 (90.79)	0–187.5 (65.52)
	Magnesium	10.0–13.3 (11.1)	6.0 (−)	6.67–9.50 (7.78)	14.2–20.4 (16.8)	2.10–14.6 (8.34)	6.67–17.1 (15.1)
	Sodium	34.6–50.4 (44.1)	36.4–65.0 (46.9)	38.3–75.0 (64.2)	4.00–100.0 (46.04)	25.0–75.0 (38.8)	0.62–62.0 (23.9)
	Potassium	132–156 (144.2)	17.0–50.0 (37.1)	23.0–79.0 (62.4)	100.0–286.0 (157.4)	15.4–83.0 (37.1)	14.58–204.58 (60.2)
	Phosphorus	48.1–101.0 (84.4)	14.7–42.0 (24.6)	8.00–21.4 (14.95)	31.1–45.0 (38.1)	7.60–62.0 (39.46)	8.33–41.0 (24.67)
Trace elements	Iron	0.02–0.05 (0.03)	0.03–0.75 (0.39)	0.08–0.30 (0.24)	0.30–0.46 (0.40)	0.01–0.45 (0.16)	0.04–2.73 (0.62)
	Copper	0.01–0.03 (0.01)	0.01 (−)	0.03 (−)	0.11 (−)	0.01 (−)	0.13 (−)
	Zinc	0.37–0.48 (0.42)	0.04 (−)	0.09–0.63 (0.24)	0.21–0.31 (0.26)	0.05–0.31 (0.18)	0.16–0.63 (0.30)
	Selenium	0.00–3.70 (2.33)	−	−	−	−	0.0–2.0 (1.50)
	Manganese	0.002 (−)	0.045 (−)	0.069 (−)	0.144 (−)	0.023 (−)	0.25 (−)
References		[8,24,26,27,69]	[8,26,29,31,69]	[8,24,26,32,69]	[8,24,26,29,33,69]	[8,24,26,34,69]	[8,24,26,35,69]

## Data Availability

Not applicable.
